# Effects of saffron (*Crocus sativus*) on sexual dysfunction among men and women: A systematic review and meta-analysis

**Published:** 2019

**Authors:** Hossein Ranjbar, Akram Ashrafizaveh

**Affiliations:** 1 *Department of nursing, School of nursing and midwifery, Torbat Heydariyeh University of Medical Sciences, Torbat Heydariyeh, Iran.*; 2 *Department of midwifery, School of nursing and midwifery, Torbat Heydariyeh University of Medical Sciences, Torbat Heydariyeh, Iran.*

**Keywords:** Saffron, Crocus sativus, Sexual, Sexual dysfunction

## Abstract

**Objective:**

This systematic review and meta-analysis study evaluated the effect of saffron (*Crocus sativus*) on sexual dysfunction and its subscales (dimensions) among men and women.

**Material and Methods:**

PubMed/Medline, ScienceDirect, Google Scholar, as well as Scientific Information Database (www.SID.ir) and Magiran (as Persian databases) were searched without any time and language restrictions. Statistical pooling was done using the random effects model.

**Results:**

A total of 5 studies comprising 173 participants were included in this systematic review and meta-analysis. The analysis showed a statistically significant positive effect of saffron on sexual dysfunction (Std diff in means=0.811; 95% CI, 0.356–1.265) and its subscales (Std diff in means=0.493; 95% CI, 0.261–0.724). Heterogeneity indexes such as Cochran Q index and $I^{2}$ indicated a heterogeneity among the included studies (Q=9:981, df:4, (p=0.041), I^2^=59.92%). There was no evidence of publication bias in these studies.

**Conclusion:**

In general, saffron was proven effective in improving sexual dysfunction and its subscales among participants; this effect was different on different dimensions of sexual dysfunction. Further studies are required to extend these initial findings.

## Introduction

Sexual dysfunction is a globally common health problem that affects the health and quality of life of the patients (Walsh and Berman, 2004 ▶). Sexual dysfunction is more common in women than men (Kotta et al., 2013 ▶; Lewis et al., 2004 ▶; Lewis et al., 2010 ▶; Moreau et al., 2016 ▶), mainly due to the physiological, anatomical and genetic differences between men and women (Costantini et al., 2017 ▶). Clinical forms of this disease in women commonly involves lack of sexual desire, impaired arousal, inability to achieve orgasm, or sexual activity with pain (Aslan and Fynes, 2008 ▶; Costantini et al., 2017 ▶; Walsh and Berman, 2004 ▶). In men, sexual dysfunction is often seen as abnormal libido, erectile function, ejaculation, orgasm, and detumescence (Laumann et al., 1999 ▶). These conditions may be the most important and primary signs and symptoms of systemic disorders that affect the health of the affected person (Minhas and Mulhall, 2017 ▶). In some studies, associations between sexual dysfunction and various illnesses such as diabetes, obesity (Bebb et al., 2018 ▶), cardiovascular problems (Vestergaard et al., 2017 ▶), mental disorders (Fanta et al., 2018 ▶), multiple sclerosis (Scheepe et al., 2017 ▶), and postpartum sexual dysfunction were reported (Khajehei et al., 2015 ▶). Hence, timely diagnosis and treatment of this disorder is of particular importance to promote the health of individuals as well as the community (Lipshultz et al., 2016 ▶). For this purpose, various therapeutic and non-pharmacological approaches are used for treatment of this disorder (Lipshultz et al., 2016 ▶; Tsai et al., 2011 ▶; Walsh and Berman, 2004 ▶). Among the promising methods, available chemical drugs have limited efficacy, unpleasant side effects, and contraindications in some specific cases (Lipshultz et al., 2016 ▶; Sumalatha et al., 2010 ▶). Herbal medicines and especially saffron, produce anti-inflammatory, anti-oxidative (Mahmoudzadeh et al., 2017 ▶), anti-cancer (Bolhassani et al., 2014 ▶), antidepressant (Hausenblas et al., 2015 ▶; Mazidi et al., 2016 ▶) and specifically sexual enhancement effects (Hosseinzadeh et al., 2008 ▶) in humans and animals as reported by some studies.

Sexual dysfunction improving effects of saffron were investigated in women and men in several studies (Hosseinzadeh et al., 2008 ▶; Kashani et al., 2013 ▶; Modabbernia et al., 2012 ▶; Mohammadzadeh-Moghadam et al., 2015 ▶; Safarinejad et al., 2010 ▶; Safarinejad et al., 2011 ▶); but, the results were controversial. 

Although, a meta-analysis demonstrated that saffron has a positive effect on erectile dysfunction in men (Maleki-saghooni et al., 2018 ▶) , but its effect on sexual dysfunction and its other dimensions such as arousal, desire, intercourse satisfaction, orgasm, and overall satisfaction in men and women, remains unknown. 

To fill the existing information gap, the present systematic review and meta-analysis was conducted to accurately evaluate the data obtained by interventional studies in terms of the effect of saffron on sexual dysfunction and its dimensions in patients.

## Materials and Methods

**Search strategy**

**Figure 1:**
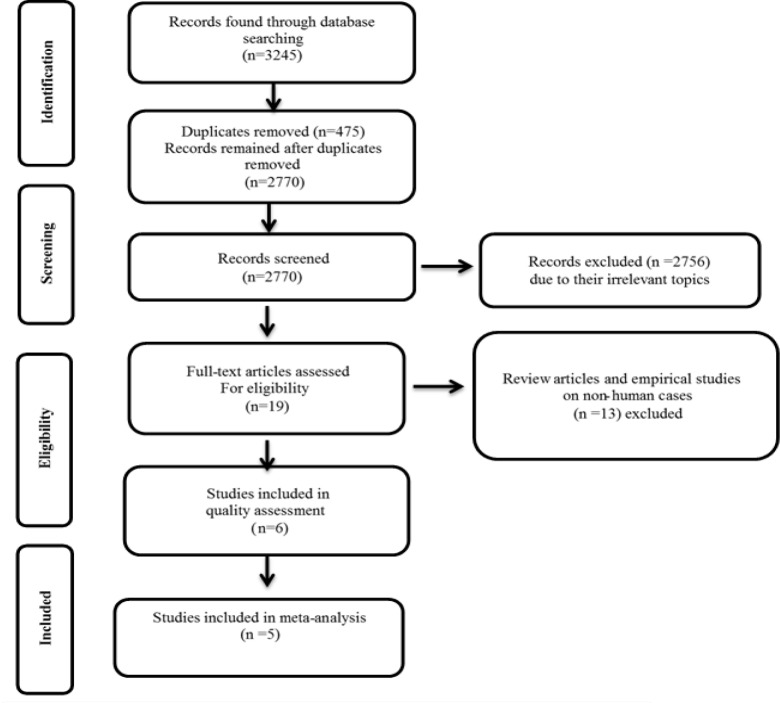
PRISMA flowchart of the study selection pricess

Two researchers individually investigated PubMed/Medline, ScienceDirect, Google Scholar, as well as Scientific Information Database (www.SID.ir) and Magiran (as Persian databases) without any time and language limitations until February 2018 using the following combination of keywords: “sexual” AND “sexual dysfunction” AND “Saffron” OR “*Crocus sativus*”. The bibliography of articles selected for meta-analysis as well as articles cited by them was also reviewed. All studies that reported the effect of saffron on sexual dysfunction in women and men and those with participants who had a mental feeling of sexual dysfunction were included in this study. Review and empirical studies on non-human cases were not included in this study. The PRISMA flowchart of the study selection process is shown in Figure1. 

**Data Extraction**

Using a pre-designed checklist, two researchers separately extracted the following relevant data from each study included in the meta-analysis: first author's name, the year of publication of the article, the name of the country, the study design, participants’ demographics (in terms of sample size, sex, gender, and age), the intervention (including the dosage form of saffron given as a treatment such as tablets or capsules, as well as dose and duration of treatment), control group (including placebo or other drugs, as well as dose and duration of treatment) as well as the main findings. Disagreement between the researchers was resolved through consensus. The characteristics of the included studies are presented in Table1.

**Quality assessment**

The quality of the selected studies was also determined by two researchers by means of a qualitative assessment tool for clinical trials ("CONSORT 2010 checklist of information to include when reporting a randomised trial"). For this purpose, articles fulfilling 75% of the criteria in the checklist, were included in this study. 

**Statistical analysis**

Data analysis was performed using a Random Effect Model. Also, $I^{2}$ index was used to determine the heterogeneity ratio among the studies included in the meta-analysis and the Cochrane Q Test. The funnel plots, the Begg and Mazumdar Rank Correlation (Duval and Tweedie, 2000 ▶) and Egger’s Regression Intercept (Egger et al., 1997 ▶) were used to determine the publication bias in these studies. Data analysis was performed using Comprehensive Meta-Analysis (Version 3).

## Results

The flowchart presented in Figure 1 shows the process of selecting studies. A total of 3,245 studies were found following a comprehensive search in PubMed/Medline, ScienceDirect, Google Scholar, and also Scientific Information Database and Magiran (as Persian databases) databases. Of these, 475 studies were excluded from the study due to repetition. In the early screening of titles and abstracts, 2756 studies were excluded due to not being of related topics. The full texts of the 19 remaining articles were thoroughly evaluated. Finally, 13 studies were excluded. In the end, 5 papers focusing on the effect of saffron on sexual dysfunction with a total of 173 participants were included in the study (Abedimanesh et al., 2017 ▶; Kashani et al., 2013 ▶; Modabbernia et al., 2012 ▶; Mohammadzadeh-Moghadam et al., 2015 ▶; Shamsa et al., 2009 ▶). 

**Table 1:** Characteristics of the 5 clinical trials included in the systematic review and meta-analysis

**Main results**	**Control group**	**Treatment group**	**Participants**	**Design**	**Country**	**First author** **(year)**
saffron had no statistically significant effect on male sexual desire	placebo capsule 30 mg/daily for 8 weeks	1- saffron capsule (Saffron Aqueous Extract) 30 mg/daily for 8 weeks 2- crocin capsule 30 mg/daily for 8 weeks	58 male and female with sexual desire disorder (mean age (year) =55.16)	RCT (three groups)	Iran	
saffron had a beneficial impact on total FSFI score and a few domains of sexual dysfunction	placebo capsule 30 mg/daily for 4 weeks	saffron capsule 30 mg/daily for 4 weeks	34 female with sexual dysfunction (mean age (year)=none available)	RCT (two group)	Iran	
saffron had positive impacts on a few domains of erectile dysfunction	placebo capsule 30 mg/daily for 4 weeks	saffron capsule 15 mg twice per day For 4 weeks	30 male with erectile dysfunction (mean age (year)=32.5)	RCT (two group)	Iran	
saffron had positive impacts on male erectile dysfunction and entire domains of erectile dysfunction	placebo gel 1% before a sexual intercourse for 4 weeks (AO)	saffron gel 1% before a sexual intercourse for 4 weeks (AO)	50 male with erectile dysfunction (mean age (year)=58.7)	RCT (two group)	Iran	
saffron had a significant impact on erectile dysfunction	-	saffron tablet 200 mg /daily for 10 days	20 male with erectile dysfunction (mean age (year)=43.78)	clinical trial (one group)	Iran	

AO=according to the order (a pea-sized amount of the gel before a sexual intercourse) Female Sexual Function Index *SFI*).

The characteristics of the included studies are presented in Table 1. By qualitative analysis of the investigated studies, the following results were obtained.

In a clinical trial study, the effect of the aqueous extract of saffron and the crocin capsule on females and males was studied using the Hulbert index of sexual desire (HISD). This study showed no significant difference between the mean scores of the group receiving saffron aqueous extract (42.10±17.55) and the group receiving crocin (37.75±15.96), and the control group (30.84±13.98) (P=0.094) (Abedimanesh et al., 2017 ▶).

In a double-blind clinical trial study, 34 women with severe depressive disorder (treated with fluoxetine for at least 6 weeks) and sexual dysfunction entered the intervention and control groups. The intervention group received saffron capsule and control group received placebo capsule daily for 4 weeks. In general, the findings indicated the beneficial effects of saffron on sexual dysfunction among women (p=0.001). Although saffron showed a statistically significant effect on some of the subscales of sexual dysfunction such as arousal (p=0.028), lubrication (p=0.035) and pain (p = 0.016), but this effect on other dimensions of sexual dysfunction scales such as desire, satisfaction and orgasm were not observed (p<0.05) (Kashani et al., 2013 ▶).

In three clinical trials, the effect of saffron on erectile dysfunction in men was studied (Modabbernia et al., 2012 ▶; Mohammadzadeh-Moghadam et al., 2015 ▶; Shamsa et al., 2009 ▶). In a randomized double-blind placebo-controlled study, 30 men with erectile dysfunction were recruited in two groups of intervention (received saffron) and control (received placebo) for 4 weeks. In summary, the findings indicated that saffron had a statistically significant effect on erectile function improvement (p=0.001), and intercourse satisfaction (p=0.001) among men, but this effect on other subscales of erectile dysfunction such as orgasm (P=0/09), sexual desire (p=0.51) and general satisfaction (p=0.33) were not observed (Modabbernia et al., 2012 ▶). In a randomized double-blind clinical trial, 50 men with erectile dysfunction were treated in two intervention groups (received saffron gel) and control (received placebo gel) for 4 weeks. In summary, the findings indicated the positive effect of saffron on the erectile function (p=0.001), as well as all subscales of the international indicators of erectile function (i.e. general satisfaction (p=0.001), intercourse satisfaction (p<0.001) orgasmic function (p<0.001), sexual desire (p<0.001), and erectile function (p<0.001)) in men (Mohammadzadeh-Moghadam et al., 2015 ▶). In a clinical trial, 20 men with erectile dysfunction were studied before and after the intervention. The status of erectile dysfunction before intervention was evaluated by a questionnaire of international indicators of erectile function before the study and then after 10-day treatment with saffron tablets. The results showed that the use of saffron tablet resulted in an improvement of erectile function (p<0.001) and all subscales including dyspepsia, sexual desire, sexual satisfaction, and general satisfaction (for all cases p<0.001) (Shamsa et al., 2009 ▶). 

**Figure 2:**
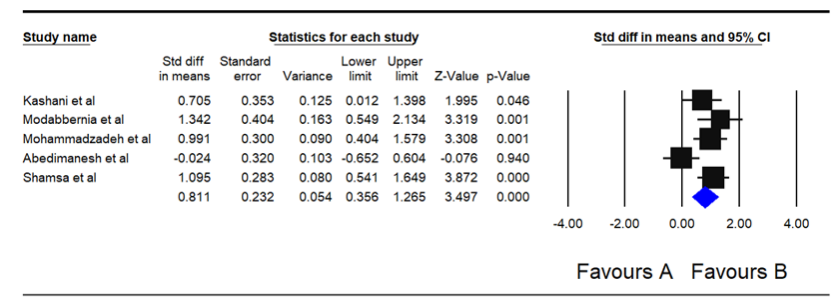
Forest plot of effect of saffron on sexual dysfunction based on the random effects model

In Figure 2, the forest plot shows the effect of saffron on sexual dysfunction based on studies included in the meta-analysis. The effects of saffron on sexual dysfunction and its subscales varied among the five papers included in the meta-analysis. In one study, saffron was ineffective with respect to sexual dysfunction (Abedimanesh et al., 2017 ▶), while it was effective in the other studies (Kashani et al., 2013 ▶; Modabbernia et al., 2012 ▶; Mohammadzadeh-Moghadam et al., 2015 ▶; Shamsa et al., 2009 ▶). Overall, the pooled results of the present work showed that saffron improved sexual dysfunction among participants (Std diff in means=0.811; 95% CI, 0.356–1.265) (Figure 2).

**Figure 3:**
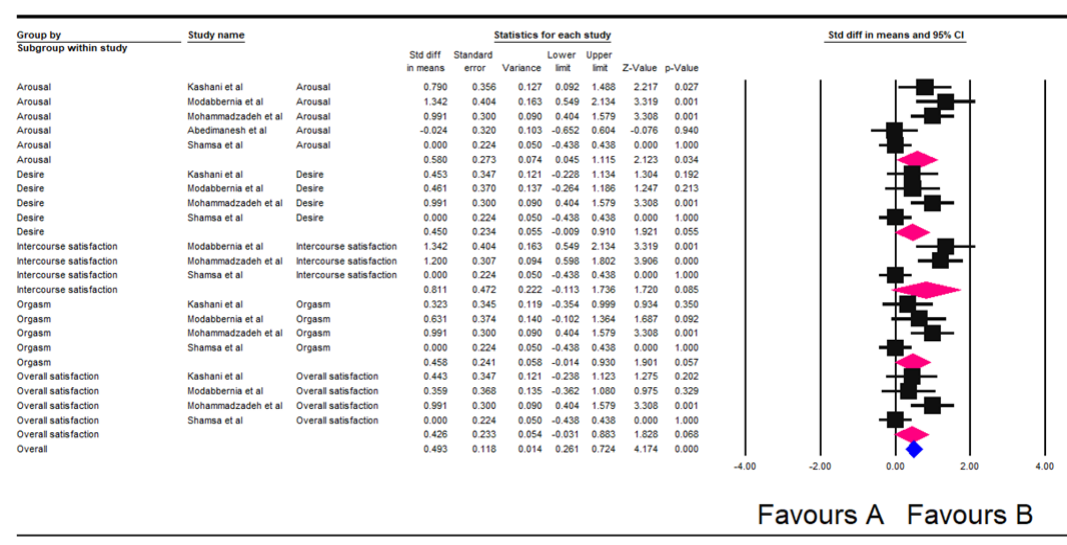
Forest plot of the effect of saffron on the sub-scales of sexual dysfunction based on the random effects model

In the subgroup analysis related to subscales of sexual dysfunction in general, the pooled results of the study indicated beneficial effect of saffron on the subscales of sexual dysfunction (Std diff in means=0.493; 95% CI, 0.261–0.724). Although, among the subscales of sexual dysfunction, saffron was effective in improving intercourse satisfaction (Std diff in means=0.811; 95% CI, -0.113-1.736), saffron was ineffective with respect to other subscales of sexual dysfunction including arousal (Std diff in means=0.580; 95% CI, 0.045-1.115), desire (Std diff in means=0.450; 95% CI, -0.009-0.910), orgasm (Std diff in means=0.458; 95% CI, -0.014-0.930) and overall satisfaction (Std diff in means=0.426; 95% CI, -0.031-0.883) (Figure 3).

**Figure 4:**
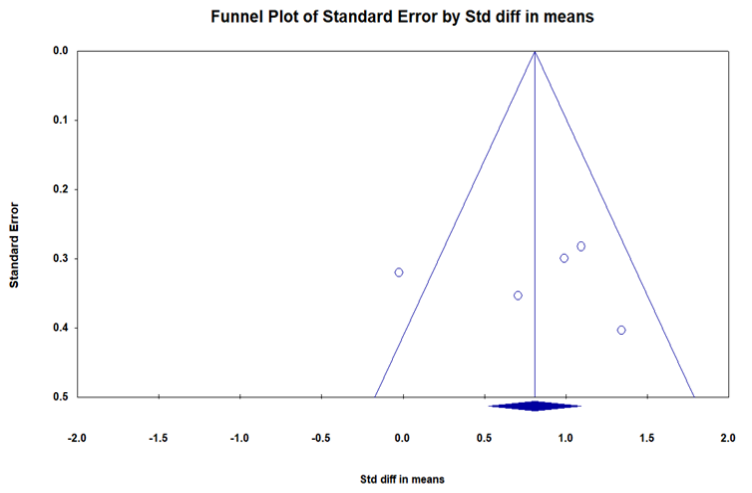
Funnel plot of publication bias of the effect of saffron on sexual dysfunction

The Cochran Q index also showed a heterogeneity of Q=9: 981, df: 4, (p=0.041) among the studies included in the meta-analysis. The index$I^{2}$=59.92% showed a moderate real heterogeneity ratio among these studies. In order to study the publication bias, Funnel plot, and also the Egger’s regression intercept and Begg and Mazumdar rank correlation were used. Figure 4 shows a funnel plot of publication bias related to the effect of saffron on sexual dysfunction among participants. Egger’s regression intercept (p=0.733) and Begg and Mazumdar rank correlation (p=0.462) indicated a relative symmetry of the Funnel plot and showed no evidence of publication bias on the effect of saffron on sexual dysfunction (Figure 4).

## Discussion

The present systematic review and meta-analysis investigated the effect of saffron on sexual dysfunction. The results showed that saffron has a statistically significant positive effect on sexual dysfunction among the patients (Std diff in means=0.811; 95% CI, 0.356–1.265). This finding is consistent previous reports (Kashani et al., 2013 ▶; Modabbernia et al., 2012 ▶; Mohammadzadeh-Moghadam et al., 2015 ▶; Shamsa et al., 2009 ▶). The results of a systematic review and meta–analysis showed that saffron has a positive effect on erectile dysfunction in men (Maleki-saghooni et al., 2018 ▶). However, in one study, saffron produced no effect in terms of improvement of sexual dysfunction (Abedimanesh et al., 2017 ▶). These differences may be due to different conditions affecting the implementation of these studies.

In the subgroup analysis related to the subscales of sexual dysfunction in general, the pooled results of the study indicated the positive effect of saffron on the improvement of sexual dysfunction in the related subscales (mean Std diff=0.493; 95% CI, 0.261–0.724). This finding is consistent with the results of a previous study which showed the effect of saffron on the improvement of sexual dysfunction in terms of erectile dysfunction, among men (Maleki-saghooni et al., 2018 ▶).

Further analysis of sexual dysfunction subscales revealed that saffron only improved sexual satisfaction, but such effect was not observed for other subscales of sexual dysfunction such as arousal, desire, orgasm and overall satisfaction. In one study, saffron was effective in treating sexual function associated with the arousal and lubrication subscales, but was ineffective for other subscales of sexual dysfunction including desire, satisfaction, and orgasm (Kashani et al., 2013 ▶). 

In the study done by Modabernia et al. (2012) ▶, saffron was effective with respect to the subscales of erectile function and intercourse satisfaction, but did not affect other subscales of sexual dysfunction. In the other studies, saffron effectively improved all subscales of sexual dysfunction (Mohammadzadeh-Moghadam et al., 2015 ▶; Shamsa et al., 2009 ▶). 

It seems that dissimilarities among the findings of the studies under discussion are likely due to differences in the individual factors of the participants, saffron consumption method, the study conditions, and the underlying illnesses of the participants enrolled into the study. Also, the present work indicated heterogeneity among the studies included in the meta-analysis. This heterogeneity seems to be due to differences in designs of the studies, type of interventions and individuals, and environmental factors governing the studies. However, due to the small number of available studies done in this field, it is not possible to investigate the effect of covariates including the nature of the intervention (type of saffron consumed, such as capsule, gel or tablet), duration of treatment, saffron consumption, saffron doses, participants gender, sexual dysfunction assessment tool and underlying illness using metastatic analysis. Further investigation of these variables should be carried out by future clinical trial studies on the subject.

In general, saffron was effective in improving sexual dysfunction and its dimensions among participants, although the significance of this effect varied in terms of different aspects of sexual dysfunction. Further studies are recommended to evaluate saffron effects on sexual dysfunction.
